# The Weak Small RNA-Binding Activity of the 2b Proteins of Subgroup II Cucumber Mosaic Virus Strains Is Insufficient for RNA Silencing Suppression

**DOI:** 10.3389/fmicb.2021.760937

**Published:** 2021-10-22

**Authors:** Yingying Gao, Jinrui Yang, Xiaobei Zhang, Aizhong Chen, Zhouhang Gu, Zhiyou Du

**Affiliations:** College of Life Sciences and Medicine, Zhejiang Sci-Tech University, Hangzhou, China

**Keywords:** cucumovirus, RNA silencing, 2b, RNA-binding activity, pathogenicity

## Abstract

The 2b proteins encoded by cucumber mosaic virus (CMV) subgroup I strains suppress RNA silencing primarily by competitively binding small RNAs (sRNAs) in the host cell cytoplasm. Interestingly, 2b proteins encoded by CMV subgroup II strains accumulate predominantly in nuclei. Here we determined that whereas the 2b protein (Fny2b) of subgroup IA strain Fny-CMV is highly effective in suppressing both sense RNA-induced and inverted repeat-induced posttranscriptional gene silencing, the 2b protein (LS2b) of the subgroup II strain LS-CMV was not as effective. Reducing nuclear accumulation of LS2b by mutating a residue in its nuclear localization sequence had no effect on RNA silencing suppressor activity, while attenuated viral symptoms. Electrophoretic mobility shift assays showed that the sRNA binding of LS2b was weaker and more selective than that of Fny2b. The domain determining the differential sRNA-binding ability was delimited to the putative helix α1 region. Moreover, LS2b mutants that completely lost suppressor activity still retained their weak sRNA-binding ability, suggesting that sRNA binding is not sufficient for LS2b to suppress RNA silencing. Considering the subgroup I strain-encoded 2b proteins that require sRNA-binding ability for the suppression of RNA silencing, we suggest that in addition to binding sRNA, the 2b proteins of subgroup II CMV strains would require extra biological activities to achieve RNA silencing inhibition.

## Introduction

RNA silencing is a conserved mechanism in eukaryotes in which RNA accumulation is regulated by sequence-specific interactions with small RNAs (sRNAs; Palukaitis, 2011). In plants, the major sRNA classes are genetically encoded microRNAs (miRNAs) that regulate endogenous RNA turnover and ensure normal plant development and *de novo*-synthesized short-interfering RNAs (siRNAs) which, among other things, provide a key defense layer against virus infection (Palukaitis, 2011). During virus infection, double-stranded structures within viral RNAs or replication intermediates are recognized as substrates by Dicer-like (DCL) endoribonucleases to produce virus-derived siRNAs (vsiRNAs), which are incorporated into argonaute (AGO) proteins, the core components of RNA-induced silencing complexes (RISCs) that degrade viral RNAs in a sequence-specific manner (Soosaar et al., 2005; Palukaitis, 2011; Csorba et al., 2015). Most plant viruses express one or more viral suppressors of RNA silencing (VSRs) to counteract siRNA-mediated antiviral defense (Roth et al., 2004; Li and Ding, 2006; Csorba et al., 2015; Li and Wang, 2019).

The VSRs of different viruses vary considerably with respect to the mechanisms employed to inhibit antiviral RNA silencing. Certain VSRs inhibit vsiRNA biogenesis by interfering with the activities of double-RNA-binding protein 4, DCLs, the host RNA-dependent RNA polymerase 6 (RDR6), or the suppressor of gene silencing 3 (SGS3). Other VSRs compromise slicing activity or stability of AGO proteins, while many VSRs sequestering vsiRNAs inhibits their incorporation into RISCs (Roth et al., 2004; Li and Ding, 2006; Csorba et al., 2015; Li and Wang, 2019). VSRs can influence viral pathogenicity in manners that are dependent or independent of their VSR activities (Diaz-Pendon and Ding, 2008; Csorba et al., 2015).

Cucumber mosaic virus (CMV) is an economically important plant pathogen which, for example, is the dominant virus infecting field-grown vegetables crops in China (Liu et al., 2019). CMV occurs worldwide and has an extremely broad host range (Palukaitis and García-Arenal, 2003). The CMV genome comprises three positive-sense, single-stranded RNAs (RNAs 1–3), which encode five viral proteins in total. Most CMV strains can be divided into the subgroups IA, IB, and II on the basis of phylogenetic analysis of the CMV coat protein (CP) gene or 3' untranslated region (Roossinck et al., 1999). Differences in virulence between subgroup I and subgroup II strains in some hosts plants can be attributed to the properties of their respective 2b proteins (Wahyuni et al., 1992; Zhang et al., 1994; Cillo et al., 2009). Additionally, the 2b proteins encoded by cucumoviruses including CMV and tomato aspermy virus (TAV) are VSRs (Lucy et al., 2000; Guo and Ding, 2002; Chen et al., 2008). The crystal structure of the C-terminally truncated TAV 2b protein (TAV2b) shows that TAV2b forms a homodimer providing the structural basis for binding sRNA duplexes (Chen et al., 2008). The mutation of any of several TAV2b residues engaged in sRNA binding strongly impairs VSR activity, suggesting that TAV2b inhibits RNA silencing by binding sRNA directly (Chen et al., 2008). The 2b proteins encoded by subgroup I CMV strains including Fny, SD, and CM95R, also sequester sRNAs and prevent their incorporation into RISC (Goto et al., 2007; González et al., 2010, 2012; Duan et al., 2012). Additionally, several independent studies have demonstrated that the 2b proteins encoded by strains of CMV subgroup I inhibit the RNA cleavage activities of AGO1 and AGO4 by direct interaction (Zhang et al., 2006; Hamera et al., 2011; Duan et al., 2012). The 2b-AGO interaction is not required for silencing suppression in agroinfiltration assays (Duan et al., 2012), while it was found to be necessary for interference with RDR1/6-dependent antiviral silencing in plants (Fang et al., 2016). Constitutive expression of the 2b proteins from subgroup I strains in transgenic Arabidopsis plants disrupts miRNA-mediated silencing, causing plant developmental defects (Zhang et al., 2006; Lewsey et al., 2007; Xu et al., 2013; Du et al., 2014a; Dong et al., 2016). However, transgenic expression of the 2b protein encoded by subgroup II stain Q or LS had little effect on Arabidopsis development, which was suggested to be due either to instability *in vivo* (Zhang et al., 2006) or to lack of a functional domain for disrupting miRNA functions (Lewsey et al., 2007). Consistent with the differential ability to disrupt miRNA function, the 2b proteins of subgroup I strains display stronger suppression of siRNA-mediated posttranscriptional gene silencing (PTGS) of a *green flurorescent protein* (*GFP*) transgene than those of subgroup II strains in agroinfiltration assays (Lucy et al., 2000; Ye et al., 2009). However, 2b proteins from a subgroup IA and a subgroup II strains are equally able to counteract self-silencing of a potato virus X-derived replicon in Arabidopsis (Lewsey et al., 2007).

When fused with a either GFP or β-glucuronidase (GUS) reporter, the 2b proteins subgroup I strains (e.g., Fny and SD) accumulate not only in nuclei but also in the cytoplasm (Wang et al., 2004; González et al., 2010; Duan et al., 2012; Du et al., 2014a), consistent with the subcellular distribution of the native Fny2b protein in CMV-infected plant cells (Mayers et al., 2000). Nuclear localization is determined by two nuclear localization signals (NLS1 and NLS2) in the 2b proteins of subgroup IA and IB CMV strains (Wang et al., 2004; González et al., 2010; Duan et al., 2012), and a single NLS in the 2b proteins of subgroup II strains. However, the 2b proteins of Subgroup II strains such as Q and LS are barely detectable in the cytoplasm, and highly enriched in nuclei (Lucy et al., 2000; Du et al., 2014a). Nemes et al. (2017) demonstrated that phosphorylation of serine residues at positions 40 and 42 occurs in the region of the 2b protein previously shown to control nuclear-cytoplasmic distribution (González et al., 2010). González et al. (2012) decreased intranuclear accumulation of Fny2b by fusing it with a nuclear export signal (NES) which did not impair its VSR activity. Together with our previous work showing that enhancing nuclear accumulation of Fny2b decreases activity (Du et al., 2014a), these results show that VSR activity occurs predominantly in the cytoplasm. However, increasing nuclear localization of Fny2b enhanced CMV virulence, indicating that nuclear localization plays an important role in 2b-mediated CMV pathogenicity (Du et al., 2014a). Since the 2b proteins of subgroup II strains (such as LS- or Q-CMV) are highly enriched in nuclei, we wondered how important nuclear localization is for the VSR and pathogenicity-determining functions of the 2b proteins of subgroup II CMV strains, and whether sRNA binding is required for their VSR activity.

## Materials and Methods

### Plant Growth

*Nicotiana benthamiana* (Domin.) plants were grown under 16-h and 8-h photoperiods, with a light intensity of 150 to 200μE.m^−2^.s^−1^ at 23–25°C. Approximately 3-week-old plants were used for virus inoculation or agroinfiltration.

### Plasmid Construction

The plasmid p35S-GFP carries a modified GFP (*mgfp5*) sequence under the control of cauliflower mosaic virus 35S promoter. The plasmids pCHF3-35S:FP and pCHF3-35S:dsFP carry a sense and inverted repeat RNA of GFP FP fragment, respectively, which were reported previously (Xiong et al., 2009; Li et al., 2014). Previously, we constructed the plasmid p35S:Fny2b that expresses Fny2b in pBI121 by the replacement of GUS with Fny2b (Xu et al., 2013). To express LS2b in pBI121, firstly LS2b gene was amplified by regular PCR and inserted into pUC18, to generate pUC18-LS2b. After authenticating pUC18-LS2b by DNA sequencing, the LS2b fragment was subcloned into pBI121 as for constructing pBI121-Fny2b (Xu et al., 2013). To construct pBI121-LS2b-derived mutants (R33A, R36A, R33/36A, P41A, R46A), site-directed mutagenesis (Jeong et al., 2012) was used to introduce the corresponding mutation in pUC18-LS2b, followed by subcloning the mutated LS2b fragment into pBI121.

The plasmids pBI121-EGFP, pBI121-EGFP-Fny2b and pBI121-EGFP-LS2b that express EGFP, EGFP-Fny2b, and EGFP-LS2b in pBI121, respectively, were constructed previously (Du et al., 2014a). To fuse a copy of PKI NES at the N terminus of EGPF-LS2b in pBI121-EGFP-LS2b, the DNA oligos NES-gfp-F and LS2b-R were used to amplify the full length of EGFP-LS2b from pBI121-EGFP-LS2b. The amplified DNA fragment was digested with *Bam*HI and *Sac*I, and then inserted into pBI121, to generate pBI121-NES-EGFP-LS2b. For generation of the plasmid pBI121-EGFP-LS2bR24Q, site-directed mutagenesis was used to introduce the mutation of R24 to Q in LS2b using pG:LS2b as the template, which harbors EGFP-LS2b in the pRTL2 plasmid (Du et al., 2014a). Then the mutated EGFP-LS2b fragment was cut off from pG:LS2b, and inserted into pBI121, generating the target plasmid.

The infectious clones pLS-CMV1, pLS-CMV2, and pLS-CMV3 corresponding to RNA1, RNA2, and RNA3 of LS-CMV have been described previously (Zhang et al., 1994). To generate the pLS209 mutant pLS2092bR24Q that expresses the mutated 2b protein harboring the mutation of arginine at position 24 by glutamine, site-directed mutagenesis was used to introduce the target mutation in pLS-CMV2. The construct pGEX-4t-Fny2b used to express GST-fused Fny2b (GST-Fny2b) in *Escherichia coli* BL21 was described previously (Du et al., 2014a). For generation of pGEX-4t-LS2b, LS2b fragment was amplified, digested by the restriction enzymes *Bam*HI and *Xho*I, and inserted into the corresponding cloning sites in the vector pGEX-4t-1. To express recombinant 2b proteins between Fny2b and LS2b, six recombinant 2b genes were synthesized artificially (Genscript) and inserted into pGEX-4t-1 as for pGEX-4t-LS2b.

### *In vitro* Transcription and Virus Inoculation

Virus inoculation was performed as previously (Du et al., 2007). Briefly, RNA transcripts corresponding to each viral genomic RNA or mutant RNA were synthesized *in vitro* from the infectious clones of LS-CMV using the T7 Ribomax *in vitro* transcription kit (Promega) in the presence of cap analogue (Promega). RNA transcripts corresponding to RNA1, RNA3, and RNA2 or mutant RNA2 were equally mixed, before inoculation on Carborundum-dusted leaves of about 3-week-old *N. benthamiana* plants.

### Agroinfiltration Assays

Agroinfiltration assays for analyses of subcellular distribution and suppressor activity were performed as described previously (Du et al., 2014a). Briefly, for analysis of subcellular distribution of LS2b and its variants, *A. tumefaciens* cells carrying the plasmid pBI121-EGFP, pBI121-LS2b or its variants were adjusted to an optical density at A_600_ of 0.25, and infiltrated into the 6th true leaves of *N. benthamiana* plants. At 3days post-infiltration, the lower epidermal cells of the infiltrated patch were imaged for GFP fluorescence by confocal scanning laser microscopy (SP5, Leica). To analyze the RNA silencing suppressor activities of Fny2b, LS2b, or 2b mutants, *A. tumefaciens* cells harboring p35S:GFP were mixed with cells carrying either 35S:FP or 35S:dsFP, together with cells carrying one of the 2b protein-expressing plasmids, or the vector pBI121 as a control, and the mixtures were infiltrated into the 5th or 6th true leaves of *N. benthamiana* plants. At 5 dpi, GFP fluorescence from the infiltrated leaves was photographed under UV illumination (Black Ray Model B 100A) using a Nikon coolpix digital camera.

### RNA Blotting

RNA (‘northern’) blot analysis of GFP mRNA was performed as described previously (Du et al., 2014a). Total RNA was extracted from agroinfiltrated leaves using TRIzol reagent (Invitrogen) according to the manufacturer’s instruction. Five micrograms of each total RNA sample was separated on 1.5% agarose gel containing 7% formaldehyde, and transferred onto Hybond+nylon membrane (GE). GFP mRNA was detected by the digoxigenin (DIG)-labeled DNA probes that were prepared using the DIG high primer DNA labeling and detection starter kit II (Roche) as recommended by the manufacturer, and cleaned up using G25 Sephadex columns (GE). DIG-labeled probes were detected using a chemiluminescence-based DIG detection kit (Roche) according to the manufacturer’s instructions.

### Immunoblotting

Immunoblot analysis of CMV CP extracted from virus-infected plants was performed according to the method described previously (Du et al., 2014a). Briefly, upper systemically-infected leaves were harvested at 14 dpi and used for extraction of total soluble proteins, which were separated in a 15% SDS–PAGE gel, and transferred onto a nitrocellulose membranes. Membranes were incubated with the polyclonal anti-CP serum (Agadia). For analyzing GFP protein, total soluble protein was extracted from leaves expressing GFP or GFP-2b fusion proteins at 5 dpi. Primary antibody binding on membranes was detected using horseradish peroxidase-conjugated anti-rabbit IgG (Santa Cruz) and ECL substrate (Thermo-Fisher).

### *In vitro* sRNA Binding of CMV 2b Proteins

Expression and purification of GST or 2b-GST fusion proteins were performed according to the procedure described previously (Du et al., 2014a). Briefly, expression of each target protein in *E. coli* BL21 cells was induced in the presence of 0.4mm IPTG (isopropyl—D-thiogalactopyranoside) at 20°C for 4h, and then purified using glutathione HiCap matrix slurry (Qiagen) following the manufacturer’s purification protocol. Purified proteins were examined by SDS–PAGE.

Just before sRNA-binding assay, the purified proteins were applied to Amicon 10K columns (Millipore) for buffer exchange, and quantified using Bradford (1976) reagent (Biorad). *In vitro* 2b-sRNA binding was done according to the procedure described previously (González et al., 2010). Here we tested three different forms of Arabidopsis miR159-, miR164-, miR166-, or miR168-derived sRNA molecules, which were synthesized commercially (Takara). These three different forms include ss-miRNA, ds-miRNA, and ds-siRNA. Ds-miRNA and ds-siRNA were generated *in vitro* as described previously (Du et al., 2014a). For instance, ds-miR168 was produced by annealing biotin-labeled miR168 with its star strand *in vitro*, and ds-siR168 was produced by annealing biotin-labeled miR168 with a 21nt sRNA that has a 19nt sequence completely complementary to miR168.

## Results

### Differential Ability of the 2b Proteins Encoded by CMV Strains in Suppression of Local GFP Silencing

**Figure 1:**
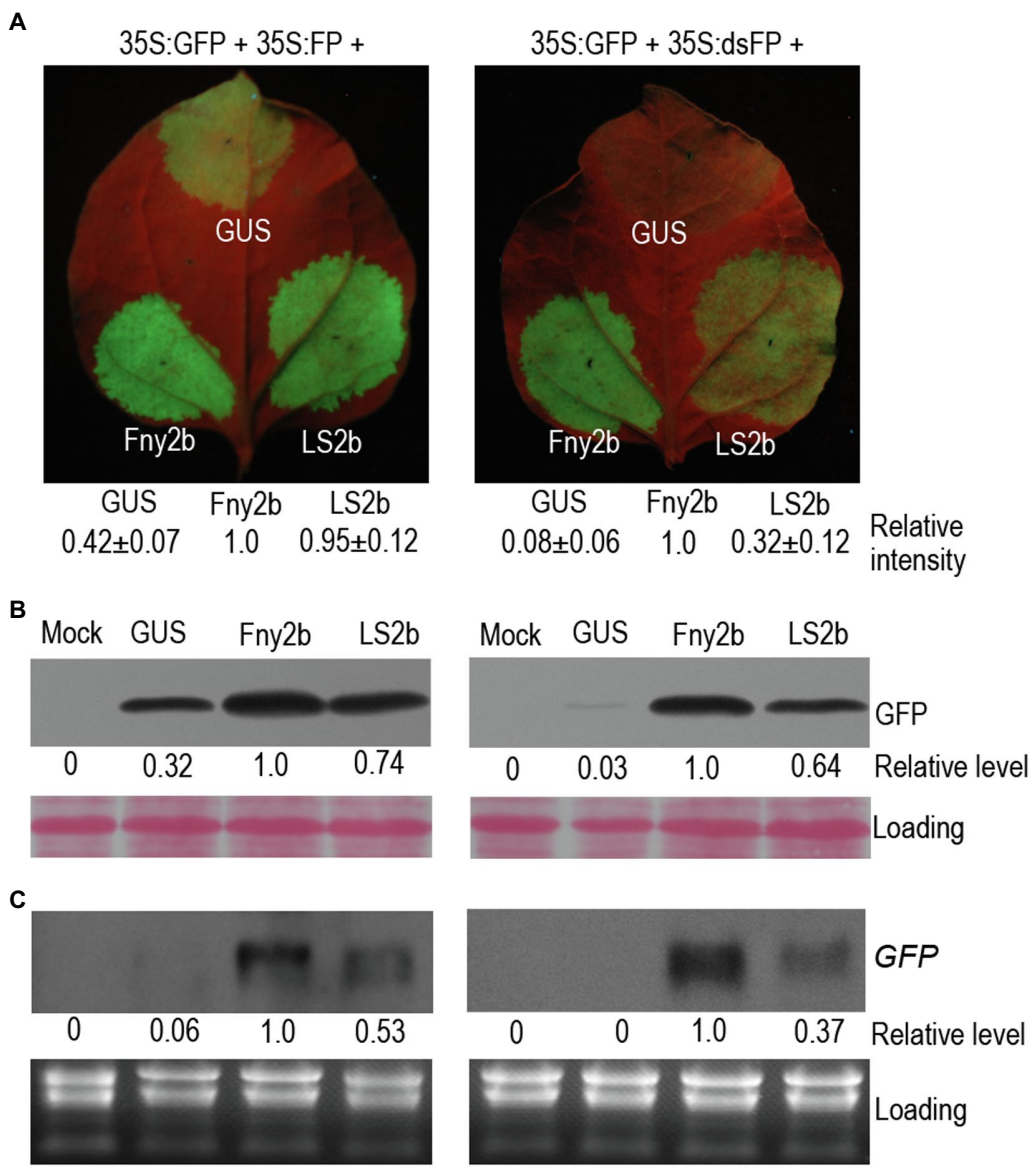
Differences in RNA silencing suppressor activity of Fny2b and LS2b in inhibition of local *GFP* silencing. **(A)** Observation of GFP fluorescence. Patches on the 6th true leaves of *Nicotiana benthamiana* plants were infiltrated with cells of *Agrobacterium tumefaciens* carrying a GFP reporter construct (35S:GFP), together with cells carrying either a construct to express an aberrant *GFP*-derived sense RNA (35S:FP; Left Panel) or a construct expressing a *GFP*-derived inverted repeat RNA (35S,dsFP; Right Panel). Co-infiltrations of these mixtures with cells carrying constructs expressing GUS (control), Fny2b, or LS2b were carried out at the positions indicated on the leaves. Agroinfiltrated leaves were photographed under UV light at 5days post-infiltration (dpi) **(A)**. GFP fluorescence intensity of each patch was quantified using ImageJ, and relative intensities were shown at bottom. The relative intensity of Fny2b was defined 1 in both agroinfiltration assays. **(B)** Immunoblot analyses of the accumulation of GFP protein in the leaf patches indicated in **(A)**. Total proteins were extracted at 5 dpi and immunoblots probed using a polyclonal anti-GFP serum. Ponceau S staining was used to confirm that equal amount of protein were loaded in each gel lane. **(C)** Northern blot analyses of the steady-state accumulation of *GFP* transcripts. *GFP* transcripts were detected using a DIG-labeled DNA probe. Ribosomal RNAs stained by ethidium bromide were used as loading controls. ‘Mock’ samples were extracted from leaf tissue infiltrated with infiltration buffer. Relative level of GFP protein **(B)** or GFP transcript **(C)** were quantified using Image J and shown at bottom. The relative level of Fny2b was defined 1 in both experiments.

To better understand VSR activity of 2b proteins encoded by CMV subgroup II strains, we compared LS2b (subgroup II) with Fny2b (subgroup IA) in suppression of local *GFP* silencing in *N. benthamiana via* agroinfiltration, using *GUS* expression as a negative control. We used two plasmid constructs to induce *GFP* silencing, 35S-FP and 35S-dsFP (Xiong et al., 2009; Li et al., 2014). 35S-FP expresses an aberrant sense RNA (FP) corresponding to the 3'-terminal 400nt of GFP that triggers sense RNA-mediated PTGS (S-PTGS), which is dependent on host RDRs to synthesize double-stranded FP (dsFP) molecules as substrates for generation of GFP siRNAs by DCLs. 35S-dsFP expresses an inverted repeat (IR) RNA of the FP fragment, which can be diced directly by DCLs to produce primary siRNAs conferring IR-PTGS. Five days after agroinfiltration, the observation of GFP fluorescence showed that in the patches expressing GUS, green fluorescence was barely observed in the presence of the inducer 35S:dsFP, while it was clearly visible in the presence of the inducer 35S:FP (Figure 1A), demonstrating that the inverted repeat RNA dsFP was more efficient than the aberrant sense RNA FP in inducing silencing of *GFP* in the agroinfiltration assay. In the presence of 35S:FP, the patches expressing LS2b displayed bright green fluorescence, which was 53% stronger than the green fluorescence in the patches expressing GUS, and was indistinguishable from the green fluorescence in the patches expressing Fny2b (Figure 1A). This indicates that LS2b and Fny2b are both effective in the suppression of FP-induced silencing of *GFP*. However, based on the rescue of GFP fluorescence, LS2b was 68% weaker than Fny2b in suppressing dsFP-induced silencing of *GFP* (Figure 1A). Consistent results of GFP fluorescence observation were obtained between LS2b and Fny2b from at least three independent experiments (data not shown). The immunoblot analysis of GFP protein (Figure 1B) and RNA blot analysis of GFP transcript accumulation (Figure 1C) showed that LS2b rescued both FP- and dsFP-induced GFP silencing 26 and 36% less than Fny2b, which is overall consistent with the observations of GFP fluorescence. These data suggest that while LS2b is an effective VSR, it is not as effective as Fny2b in suppression of both S-PTGS and IR-PTGS.

### Decreasing Nuclear Enrichment of LS2b Had Limited Effect on Its Suppressor Activity

**Figure 2:**
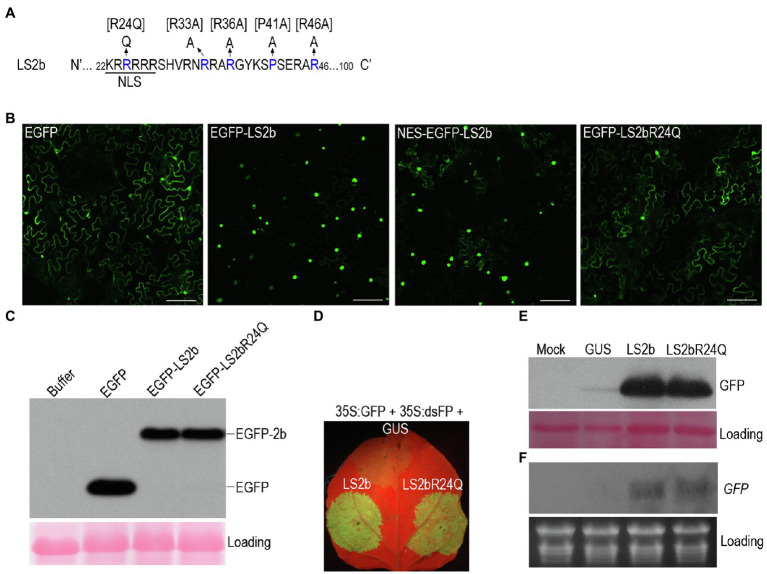
Subcellular distribution and suppressor activity of LS2b and its variants. **(A)** Partial sequence of the LS2b protein showing the nuclear localization signal and its mutant R24Q. Additional mutants bracketed were determined for their suppressor activity as shown in Figure 6. **(B)** GFP fluorescent in the epidemic cells *of N. benthamiana* plants transiently expressing free enhanced GFP (EGFP), or EGFP fusion proteins of LS2b and its mutant R24Q, namely EGFP-LS2b or EGFP-LS2bR24Q, respectively. NES-EGFP-LS2b is the variant of EGFP-LS2b that contains a nuclear exportation signal at its N terminus. GFP fluorescence was visualized at 3days post-infiltration (dpi) by confocal scanning laser microscopy. Bars show 2μm. **(C)** Immunoblot analysis of the stability of EGFP-LS2b and EGFP-LS2bR24Q using a polyclonal serum against GFP. The protein samples were prepared from the leaf tissues at 3 dpi. Ponceau S staining was used to show equal amount of total proteins loaded. **(D–F)** Comparison of suppressor activity of LS2b and LS2bR24Q in agroinfiltration assays. The 6th true leaves of *N. benthamiana* plants were infiltrated with agrobacterium cells to express GFP reporter (35S:GFP) and an inverted repeat RNA of GFP (35S:dsFP) as a silencing inducer, together with GUS, LS2b or LS2bR24Q as indicated. **(D)** GFP fluorescence produced from the agroinfiltrated leaves were photographed under UV light at 5 dpi. **(E)** Immunoblot analysis of GFP protein accumulation. A polyclonal anti-GFP serum was used to probe GFP protein. Ponceau S staining was used to monitor the relative amount of total proteins loaded. **(F)** RNA gel blot analysis of steady-state accumulation of GFP transcripts. GFP transcripts were detected using the DIG-labeled DNA probe that was made using the DIG high primer DNA labeling and detection starter kit II. Ribosomal RNAs stained by ethidium bromide were used as a loading control.

Our previous work showed that GFP-fused LS2b (GFP-LS2b) enriches in the nucleus, when transiently expressed in the leaves of *N. benthamiana via* agroinfiltration (Du et al., 2014a). To determine how important the nuclear enrichment is for LS2b to inhibit RNA silencing, GFP-Fny2b was N-terminally fused with a nuclear exportation signal (NES) that was reported to be efficient to export GFP-Fny2b from the nucleus to the cytoplasm (González et al., 2012). However, we found that the NES was insufficient to export GFP-LS2b, since NES-GFP-LS2b showed the same nuclear enrichment as GFP-LS2b (Figure 2B). The failure to alter the subcellular distribution of GFP-LS2b could be the reason that LS2b NLS1 effectively imports the exported NES-GFP-LS2b back to nucleus.

Lucy et al. (2000) reported that substitution of the residue arginine (R) at position 24 (the third residue of NLS1) in Q2b with glutamine (Q) markedly reduced the nuclear enrichment, accompanied with increased cytoplasmic distribution. Our previous work showed that substitution of the residue Q at position 24 with R in Fny2b had no discernable effect on suppressor activity of Fny2b (Du et al., 2014a). Thus, to alter the subcellular distribution of LS2b, we generated the mutant LS2bR24Q, in which the residue R24 is mutated to Q in LS2b (Figure 2A). Enhanced GFP (EGFP)-fused LS2bR24Q (EGFP-LS2bR24Q) transiently expressed in the leaves of *N. benthamiana* displayed obvious cytoplasmic distribution, but with reduced accumulation in nucleus than GFP-LS2b (Figure 2B). This demonstrates that the mutation of R24 to Q effectively weakened the nuclear importation of LS2b, leading to increased cytoplasmic accumulation, which is consistent with subcellular distribution of the 2bQGFP mutant previously reported (Lucy et al., 2000). Immunoblot analysis showed that EGFP-LS2bR24Q and EGFP-LS2b accumulate to similar levels in leaf tissue (Figure 2C), demonstrating that altering the subcellular distribution of LS2b did not alter its stability *in vivo*.

Then, we compared the VSR activities of LS2b and its mutant LS2bR24Q using agroinfiltration assays, where 35S:dsFP was used to induce IR-PTGS against *GFP* accumulation. No discernable difference was observed between the VSR activity exhibited by LS2b or its LS2bR24Q mutant, based on the intensity of GFP fluorescence (Figure 2D), or the accumulation of either the GFP protein (Figure 2E) or its transcript (Figure 2F). This suggests that nuclear enrichment is unlikely to be essential for inhibition of IR-PTGS by LS2b.

### Decreasing Nuclear Enrichment of LS2b Weakened CMV Pathogenicity in Plants

**Figure 3:**
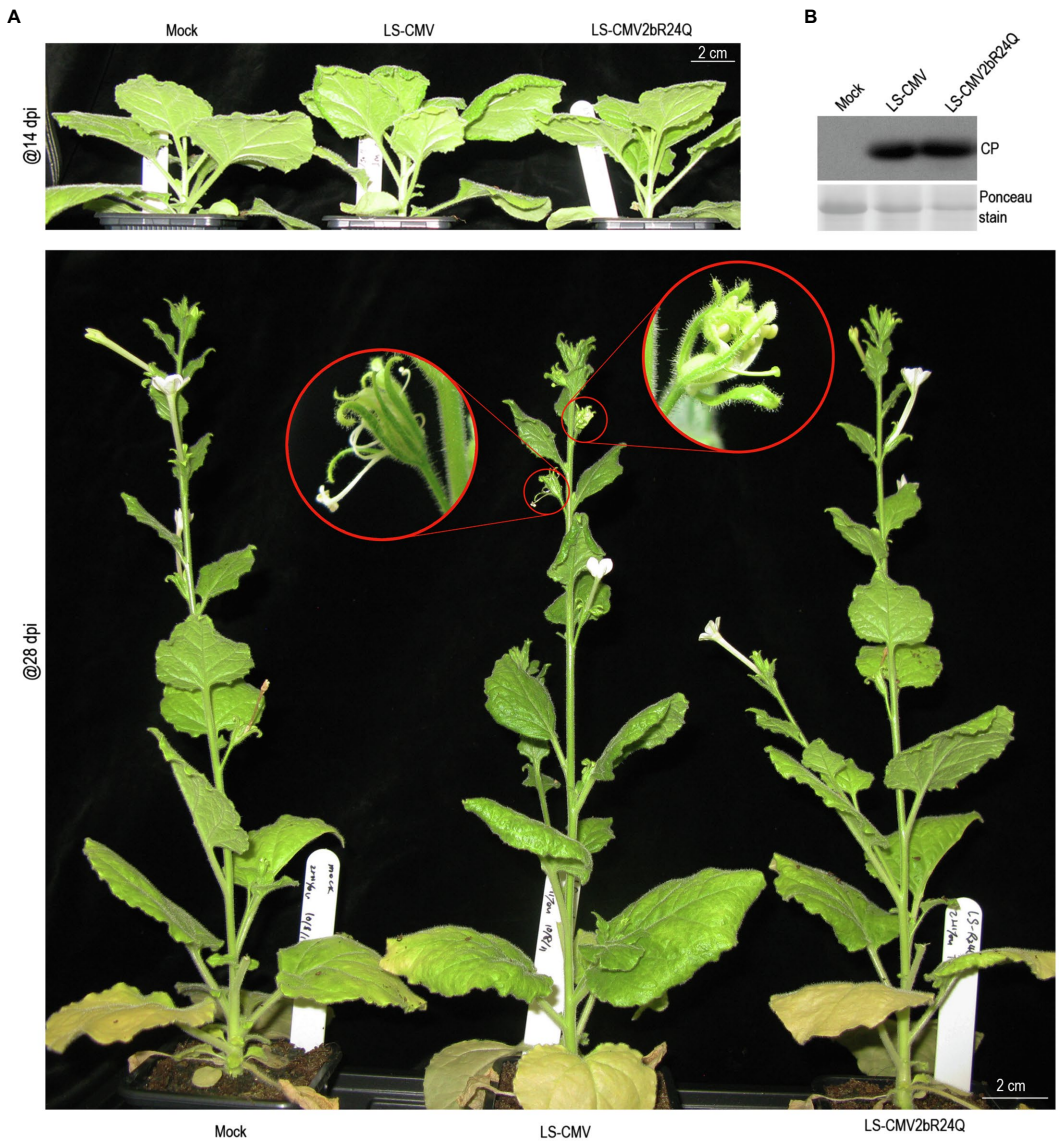
Reducing nuclear accumulation of the LS2b protein compromised viral pathogenicity in plants. **(A)** Viral symptoms on *N. benthamiana* plants inoculated with LS-CMV or its mutant LS-CMV2bR24Q. Mock is the plants treated with distilled water. The inoculated plants were photographed at 14 (upper panel) or 28 (lower panel) days post-inoculation (dpi) as indicated. The phenotypically defective flowers were shown with a close-up view as indicated by red circles (lower panel). **(B)** Immunoblot analysis of the accumulation of LS-CMV CP in the upper systemic leaves as indicated. Ponceau S staining was used to show equal amount of the protein samples loaded.

Increasing the accumulation of Fny2b in nuclei enhances Fny-CMV pathogenicity in Arabidopsis plants, an effect that is independent of interference with sRNA pathways (Du et al., 2014a). To determine whether the nuclear enrichment of LS2b influences viral pathogenicity we compared the effects of wild-type LS-CMV and a mutant (LS-CMV2bR24Q) expressing LS2bR24Q in *N. benthamiana*. It is worth mentioning that R24 was mutated to Alanine by replacing the nucleotides UGC at positions 2,841–2,843 in RNA2 with ACG that did not alter the amino acid sequence of the 2a protein. As reported previously, LS-CMV is an attenuated strain causing mild disease symptoms on the host plants Arabidopsis, tobacco and tomato (Zhang et al., 1994; Cillo et al., 2009; Du et al., 2014b). Infection by LS-CMV also caused mild symptoms on *N. benthamiana* plants, showing mild downward curling of the leaf edge (Figure 3A, upper), which began to occur about 7days after infection, and developmental defects of some flowers, including narrowed sepals, shortened petals, and irregular growth of stamens (Figure 3A, lower). However, such viral symptoms were not observed on the LS-CMV2bR24Q-infected plants that grew as well as the mock plants (Figure 3A). Immunoblot analysis showed that there was no discernable difference in the accumulation level of the viral CP protein in the upper systemic leaves with infection of LS-CMV or its mutant (Figure 3B). Our data suggest that although nuclear enrichment of LS2b has some minor effects on symptom expression, it does not have any major effect on viral titer in *N. benthamiana* plants.

### LS2b Has a Poor Ability to Bind Various sRNAs *in vitro*

**Figure 4:**
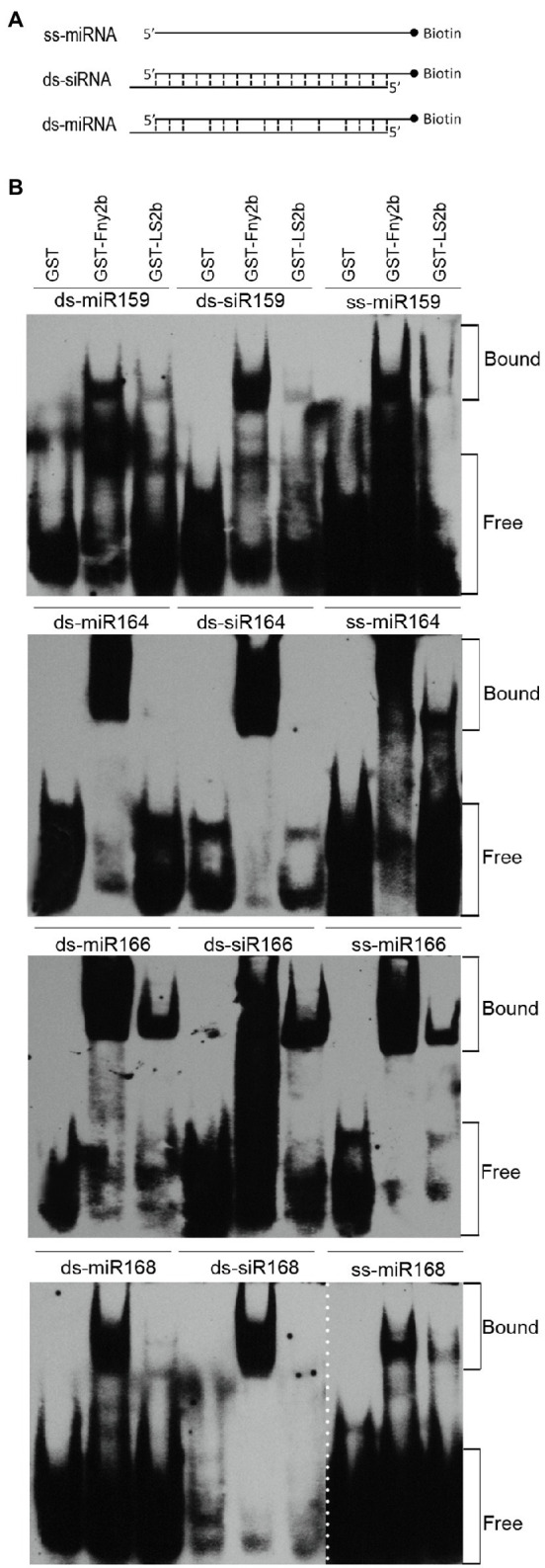
LS2b was substantially weaker than Fny2b in binding small RNAs (sRNAs) *in vitro*. **(A)** Schematic diagram of three different forms of sRNAs. Single stranded miRNA (ss-miRNA), miRNA duplex (ds-miRNA) and siRNA duplex (ds-siRNA) of four Arabidopsis microRNAs (miR159, miR164, miR166, miR168) commercially synthesized were used in the assay as FIGURE 4 | indicated in **(B)**. **(B)** Electrophoretic mobility shift assays were conducted to assess the relative sRNA-binding ability of free GST and GST-fused LS2b or Fny2b. These proteins were expressed and purified from *Escherichia coli* BL21. Biotin-labeled sRNAs were detected using horseradish peroxidase-conjugated streptavidin. The positions of bands corresponding to free and protein-bound RNAs are indicated on the right. The dash line indicates the result of ss-miR168 was not from the same gel for detection of ds-miR168 and ds-siR168.

The 2b proteins encoded by CMV subgroup I strains can bind sRNAs, and this is thought to be the primary mechanism underpinning their VSR activity (Goto et al., 2007; Duan et al., 2012; González et al., 2012). However, RNA-binding assays have not been reported for the 2b proteins of subgroup II strains, so we conducted *in vitro* sRNA-binding assays to compare the relative sRNA-binding capabilities of GST-tagged LS2b (GST-LS2b), and GST-Fny2b. In this assay, the purified GST, GST-LS2b, or GST-Fny2b was co-incubated separately with Arabidopsis miRNAs using synthetic miR159-, miR164-, miR166-, and miR168-based, miRNA duplexes (ds-miRNA), or siRNA duplexes (ds-siRNA), or single-stranded miRNA (ss-miRNA; Figure 4A). Protein-sRNA binding was detected by an electrophoretic mobility shift assay (EMSA). As reported previously (Du et al., 2014a), GST had no sRNA-binding activity, and GST-Fny2b bound to all of the sRNA molecules tested (Figure 4B).

The interactions of GST-LS2b with these four miRNA-based sRNAs was distinctly different from that of GST-Fny2b. Whereas Fny2b was able to retard the gel migration of all of these synthetic RNAs, LS2b appeared not to interact at all with miR164-derived duplexes and only with its single-stranded derivative, and LS2b gave comparable results with miR168-derived synthetic RNAs (Figure 4B). Although LS2b interacted with all three miR159-derived synthetic RNAs, the binding was markedly less strong than for Fny2b. With all three forms of the miR166-based sRNAs, GST-LS2b produced the strongest gel shift activity (Figure 4B). These EMSA data strongly suggest that either LS2b is a weak sRNA-binding protein compared to Fny2b, or that it is more selective.

### Comparison of sRNA-Binding Sites in LS2b and Fny2b

**Figure 5:**
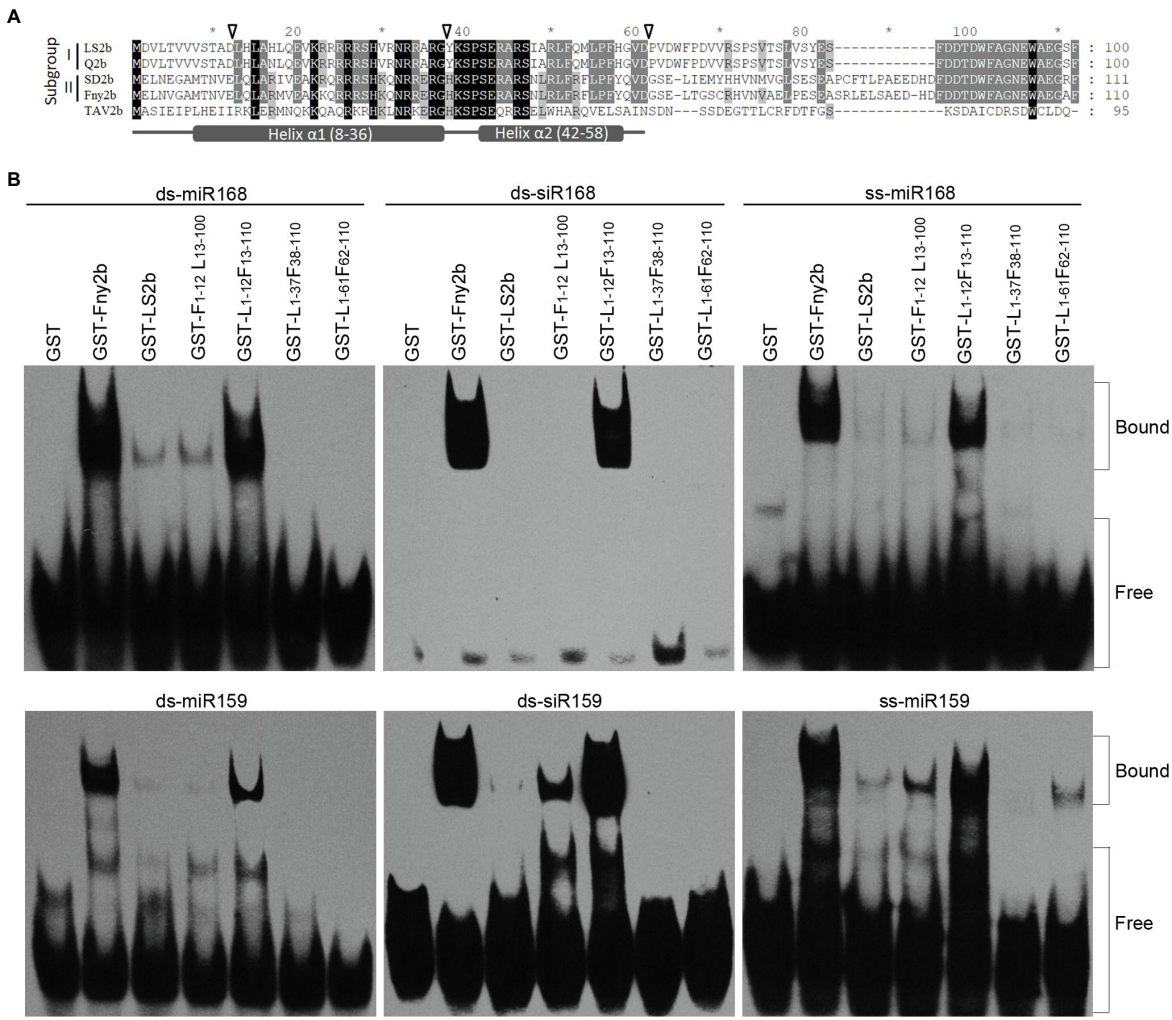
*In vitro* sRNA binding assay of GST-fused Fny2b, LS2b and their recombinants. **(A)** Alignment of 2b protein sequences of Subgroups I and II strains of CMV, as well as TAV2b. The arrows shown above the sequences indicate the exchange positions of the 2b recombinants between Fny2b and LS2b. Asterisks (*) shown above the sequences indicate intervals of every 10 residues. Putative helices α1 and α2 were shown at the bottom. **(B)** Electrophoretic mobility shift assays were conducted to assess the relative sRNA-binding ability of the GST-fused 2b proteins. The 2b recombinants were named by the formula F_1-x_L_y-100_ or L_1-x_F_y-110_, where L and F represent LS2b and Fny2b, respectively, and the lower-cased characters (x and y) represent the exchanging positions in LS2b (L) or Fny2b (F). Arabidopsis miR168 or miR159-derived ss-miRNA, ds-miRNA and ds-siRNA were commercially synthesized and used in the assay as indicated. Biotin-labeled sRNAs were detected using horseradish peroxidase-conjugated streptavidin. The positions of bands corresponding to free and protein-bound RNAs are indicated on the right.

Duan et al. (2012) determined the sRNA-binding region of the SD2b protein to be the N-terminal 61 residues, which is consistent with that of the TAV2b protein (Chen et al., 2008). The RNA-binding domain of TAV2b has two alpha helices, which are helix α1 ranging from positions 8–36 and helix α2 ranging from positions 42–58 (Chen et al., 2008; Figure 5A). Both helices are engaged in sRNA binding (Chen et al., 2008). Sequence alignment shows that in the sRNA-binding domain, only one and two residues are different between LS2b and Q2b, and between Fny2b and SD2b, respectively, but there are 27 or 28 residues difference between Fny2b or SD2b and LS2b or Q2b (Figure 5A), suggesting that the difference in the effectiveness of sRNA binding between LS2b and Fny2b (Figure 4B) is determined by the residue differences in this corresponding regions of Subgroups I and II 2b proteins. To better understand the difference between LS2b and Fny2b, we constructed six 2b recombinants between Fny2b and LS2b by exchanging their coding sequence at the positions corresponding to the residue 12, 37 or 61 as indicated by the arrows (Figure 5A). The 2b recombinants were named as L_1-x_F_y-110_ or F_1-x_L_y-100_, where L and F represent LS2b and Fny2b, respectively, and the subscript characters x and y represent the exchanging positions in LS2b (L) or Fny2b (F). These 2b recombinants with GST fusion were expressed and purified from *E. coli*. Unfortunately, two of the 6 recombinants F_1-37_L_38-100_ and F_1-61_L_62-100_ were unsuccessfully expressed probably due to cellular toxicity (data not shown). The remaining four recombinants were tested to bind three forms of miR168 or miR159-derived sRNAs as tested in Figure 4B. It is worth mentioning here that we chose miR168 and miR159-derived sRNAs because GST-Fny2b and GST-LS2b had substantial differences in binding both miRNA-derived sRNAs (Figure 4B). GST and either GST-tagged Fny2b or LS2b were included as controls in this assay. GST-F_1-12_L_13-100_ showed poor binding to all the forms of miR168- or miR159-derived sRNAs, which resembled GST-LS2b (Figure 5B). However, its reciprocal recombinant GST-L_1-12_F_13-110_ had a similarly strong ability to GST-Fny2b in binding all the forms of miR168- or miR159-derived sRNAs (Figure 5B). These results demonstrate that the poor sRNA-binding ability of LS2b is not caused by its N terminal 12 residues. Interestingly, both recombinants GST-L_1-37_F_38-110_ and GST-L_1-61_F_62-110_ exhibited extremely low binding to all the forms of these sRNAs, with the exception of GST-L_1-61_F_62-110_, which showed limited binding to ss-miR159 (Figure 5B). These data demonstrate that the differential residues in the putative helix α1 domain determine the distinct sRNA-binding ability between Fny2b and LS2b.

### Small RNA Binding Is Not Sufficient for LS2b to Inhibit Local RNA Silencing

**Figure 6:**
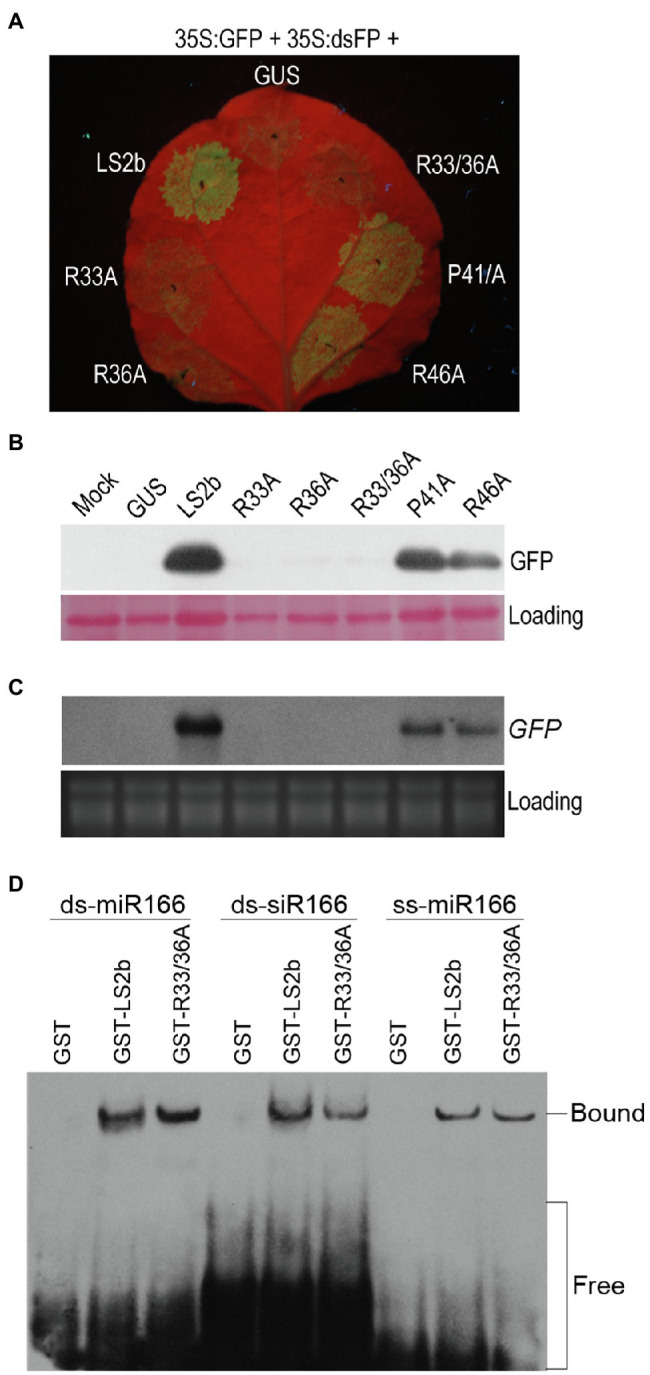
Determination of suppressor activity and sRNA binding ability for LS2b mutants. **(A)** Observation of GFP fluorescence. The 6th true leaves of *N. benthamiana* plants were infiltrated with cells of *Agrobacterium tumefaciens* harboring 35S:GFP and 35S:dsFP, together with cells expressing GUS, LS2b or its mutants as indicated. The mutants are shown in Figure 2A. R33/36A is the mutant that contains two mutations R33A and R36A. The agroinfiltrated leaves were photographed under UV light at 5days post-infiltration (dpi). **(B)** Immunoblot analysis of GFP protein accumulation in the patches as shown in **(A)**. Total proteins were prepared at 5 dpi, and detected by a polyclonal anti-GFP serum. Ponceau S staining was used to monitor the relative amount of total proteins loaded. **(C)** Northern blot analysis of the steady-stage levels of GFP transcripts. GFP transcripts were detected by the DIG-labeled DNA probe that was made using the DIG high primer DNA labeling and detection starter kit II. Ribosomal RNAs stained FIGURE 6 | by ethidium bromide were used as loading controls. **(D)** *In vitro* sRNA binding assay of LS2b and its mutant R33/36A. Arabidopsis miR166-based ss-miRNA (ss-miR166), ds-miRNA (ds-miR166) and ds-siRNA (ds-siR166) were commercially synthesized and used in the assay as indicated. Biotin-labeled sRNAs were detected using horseradish peroxidase-conjugated streptavidin. The positions of bands corresponding to free and protein-bound RNAs are indicated on the right.

So far, it seems that the relatively weak suppressor activity of LS2b is apparently correlated with its poor sRNA-binding ability, but we did not know yet whether sRNA binding is important for LS2b to inhibit RNA silencing. To this end, we attempted firstly to explore LS2b mutants that largely or completely lost VSR activity. Four residues (R33, R36, R46, and P41) were selected for individual mutation to alanine (A) to generate their corresponding mutants R33A, R36A, P41A, and R46A (Figure 2A). In addition, both R33 and R36 were simultaneously replaced with alanine to generate the double mutant R33/36A (Figure 2A). All of these four residues are completely conserved in the 2b proteins of CMV and TAV, and were previously reported to be engaged in sRNA binding in TAV2b or CMV2b (Goto et al., 2007; Chen et al., 2008). VSR activities of these mutants were tested in suppression of dsFP-induced GFP silencing *via* agroinfiltration assays. The observation of GFP fluorescence showed that green fluorescence was barely observed in the patches transiently expressing R33A, R36A or their double-mutant R33/36A, while the patches with transient expression of R46A or P41A displayed obvious green fluorescence, which was slightly weaker than that produced by LS2b (Figure 6A). The data obtained from immunoblot analysis of GFP protein (Figure 6B) and RNA blot analysis of GFP transcript (Figure 6C) in the patches were in agreement with the observation of GFP fluorescence.

Then, the double-mutant R33/36A lacking suppressor activity was selected to test its binding ability to three different forms of miR166-based sRNAs in comparison with the wild-type LS2b. It is worth mentioning here that we chose miR166-based sRNAs in this assay because LS2b had a moderate binding ability to these sRNAs (Figure 4B). EMSA assays showed that the GST-tagged mutant (GST-R33/36A) had an equivalent ability as GST-LS2b in binding all the forms of these sRNAs (Figure 6D), suggesting that sRNA binding is not sufficient for LS2b functioning as a VSR.

## Discussion

In this report, we investigated biological relevance of LS2b nuclear enrichment in RNA silencing suppression and viral symptom expression, and tested the sRNA-binding ability of LS2b and its association with VSR activity. Our results indicate that although LS2b is not as strong as Fny2b, it is effective in suppressing both S-PTGS and IR-PTGS. Nuclear enrichment contributes to symptom expression, but is dispensable for its VSR activity. Unlike Fny2b, LS2b is a weak sRNA-binding protein, and sRNA binding is insufficient for its VSR activity. Considering that the 2b proteins of Subgroup I CMV strains require sRNA binding for inhibition of RNA silencing, we suggest that in addition to binding sRNA, the 2b proteins of Subgroup II CMV strains would require extra biological activities to achieve RNA-silencing suppression.

Our data show that LS2b is much weaker in binding Arabidopsis ss-miRNAs and miRNA duplexes *in vitro* than Fny2b (Figure 4). To our knowledge, this is the first investigation of the sRNA-binding ability of 2b proteins of Subgroup II CMV strains. The difference in miRNA binding may help to explain why constitutive expression of Fny2b, but neither LS2b nor Q2b disrupts miRNA functions, leading to phenotypic defects in transgenic Arabidopsis (Zhang et al., 2006; Lewsey et al., 2007). Interestingly, LS2b seems more selective in binding these miRNA-derived sRNAs than Fny2b (Figure 4). For instance, LS2b binds miR164-derived miRNA and siRNA duplexes poorly, but single stranded miR164 and all of miR66-derived molecules efficiently. Sequence analysis shows no difference in the 5'-terminal nucleotide (Uridylate) and the sequence length (21nt) of these four Arabidopsis miRNAs tested. There is no correlation between the binding ability of these sRNAs with LS2b and the GC content of these various sRNAs. These suggest that LS2b might have a preference to sequence composition for its selectivity. More interestingly, miR159-derived molecules are bound efficiently by Fny2b, but only weakly by LS2b which, taking into account our previous work showing the importance of miR159 in symptom induction by the severe strain Fny-CMV, may help to explain why the LS-CMV symptoms are mild, while those of Fny-CMV are severe. Another interesting point is the flower developmental abnormality caused by the infection of LS-CMV in *N. benthamiana* (Figure 3). Considering that flower development is highly regulated by some miRNAs (Nag and Jack, 2010), we speculate that during viral infection, LS2b selectively binds to flower development-regulating miRNA(s) and disrupts its regulatory activity, leading to the phenotypic defects.

Our result indicates that LS2b is an effective VSR, although it is weaker than Fny2b in suppression of S-PTGS and IR-PTGS (Figure 1). This result is consistent with the previous reports that Fny2b as well as SD2b has stronger VSR activity than Q2b tested by agroinfiltration assays (Lucy et al., 2000; Ye et al., 2009). Our previous work shows that the aggregation of Fny2b into nuclei resembling the subcellular distribution of LS2b impairs its VSR activity (Du et al., 2014a). This is consistent with other VSR molecules, such as potyviral HC-Pro or tombusviral P19 that counteract RNA silencing in the cytoplasm (Riedel et al., 1998; Ye et al., 2003; Uhrig et al., 2004; Canto et al., 2006; Lakatos et al., 2006). Thus, we suggested that the relatively weak suppressor activities of 2b proteins of Subgroup II strains might be due to their more appreciable nuclear accumulation (Du et al., 2014a). However, reducing nuclear accumulation of LS2b with increased cytoplasmic distribution has no effects on its VSR activity (Figure 2). This does not support our previous suggestion, and indicates that nuclear enrichment of LS2b is dispensable for its VSR activity, which is consistent with the previous finding that Fny2b does not require its nuclear retention for suppression of RNA silencing (González et al., 2012).

The weak sRNA-binding ability of LS2b could be an explanation for its relative weak VSR activity (Figures 4, 6). However, the explanation seems contradict to the experiment with the mutant R33/36A which retains the sRNA-binding ability, while losing its VSR activity (Figure 6). Moreover, both R46 and P41, which are conserved in the 2b protein of CMV and TAV, are engaged in sRNA binding, and their mutation to alanine in CMV 2b or TAV2b is deleterious to their VSR activity (Goto et al., 2007; Chen et al., 2008), while such mutations in LS2b just shows mild effects (Figure 6). All of these mutant results suggest that sRNA binding is not sufficient, or may be dispensable for LS2b to achieve RNA silencing suppression. The 2b proteins (SD2b and Fny2b) have been determined previously to have the ability to binding long dsRNA (55bp dsRNA; Duan et al., 2012; Dong et al., 2016; Fang et al., 2016). Thus, we cannot rule out the possibility of the requirement of long dsRNA binding for LS2b to inhibit RNA silencing. Q2b dramatically reduces vsiRNA accumulation during virus infection (Diaz-Pendon et al., 2007). Wang et al. (2010) dissected the requirement of RDR-dependent secondary siRNA amplification in the siRNA-mediated anti-CMV immunity. Fang et al. (2016) determined the biological significance of SD2b-AGO interaction in production of RDR-dependent secondary siRNAs and antiviral RNA silencing. Given that Q2b has the ability to bind AGO1 and weaken the later slicer activity (Zhang et al., 2006). Thus, it is possible for the 2b proteins of Subgroup II CMV strains to inhibit RNA silencing by interacting with AGO proteins, in addition to sRNA binding. Very recently, Wang’s group identified a small peptide-encoding Arabidopsis transcript (VISP1) that is induced in Arabidopsis with CMV infection, and reduces vsiRNA biogenesis by mediating autophagic degradation of SGS3/RDR6 bodies (Tong et al., 2021). Thus, it would be interesting to determine whether 2b proteins of subgroup II CMV strains require induction of VISP1 and subsequent degradation of SGS3/RDR6 to fulfill its VSR function.

## Data Availability Statement

The original contributions presented in the study are included in the article/supplementary material, further inquiries can be directed to the corresponding authors.

## Author Contributions

ZG and ZD conceived and designed the experiments. YG, JY, XZ, and AZ performed the experiments. YG and JY analyzed the data. YG and ZG wrote the paper. ZD revised the paper. All authors contributed to the article and approved the submitted version.

## Funding

The work was supported by the National Natural Science Foundation of China (31170141 and 31870144).

## Conflict of Interest

The authors declare that the research was conducted in the absence of any commercial or financial relationships that could be construed as a potential conflict of interest.

## Publisher’s Note

All claims expressed in this article are solely those of the authors and do not necessarily represent those of their affiliated organizations, or those of the publisher, the editors and the reviewers. Any product that may be evaluated in this article, or claim that may be made by its manufacturer, is not guaranteed or endorsed by the publisher.
